# Heterogeneous factors influence social cognition across diverse settings in brain health and age-related diseases

**DOI:** 10.21203/rs.3.rs-3007086/v1

**Published:** 2023-06-09

**Authors:** Sol Fittipaldi, Agustina Legaz, Marcelo Maito, Hernan Hernandez, Florencia Altschuler, Veronica Canziani, Sebastian Moguilner, Claire Gillan, Josefina Castillo, Patricia Lillo, Nilton Custodio, José Avila-Funes, Juan Cardona, Andrea Slachevsky, Fernando Henriquez, Matias Fraile-Vazquez, Leonardo Cruz de Souza, Barbara Borroni, Michael Hornberger, Francisco Lopera, Hernando Santamaria-Garcia, Diana Matallana, Pablo Reyes, Cecilia Gonzalez-Campo, Maxime Bertoux, Agustin Ibanez

**Affiliations:** Global Brain Health Institute (GBHI); Cognitive Neuroscience Center (CNC); Cognitive Neuroscience Center (CNC); BrainLat; Cognitive Neuroscience Center (CNC); Cognitive Neuroscience Center (CNC); Latin American Brain Health Institute (BrainLat); Global Brain Health Institute (GBHI); Latin American Brain Health Institute (BrainLat); Universidad de Chile; Peruvian Institute of Neurosciences; Instituto Nacional de Ciencias Médicas y Nutrición Salvador Zubirán; Universidad del Valle; Universidad de Chile; Universidad de Chile; Cognitive Neuroscience Center (CNC); Universidade Federal de Minas Gerais (UFMG); University of Brescia; University of East Anglia; University of Antioquia; Global Brain Health Institute (GBHI); Pontificia Universidad Javeriana; Latin American Brain Health Institute (BrainLat); Cognitive Neuroscience Center (CNC); Lille Neuroscience & Cognition; Trinity College Dublin

**Keywords:** Emotion recognition, mentalizing, diverse populations, fMRI, predictive model

## Abstract

Aging may diminish social cognition, which is crucial for interaction with others, and significant changes in this capacity can indicate pathological processes like dementia. However, the extent to which non-specific factors explain variability in social cognition performance, especially among older adults and in global settings, remains unknown. A computational approach assessed combined heterogeneous contributors to social cognition in a diverse sample of 1063 older adults from 9 countries. Support vector regressions predicted the performance in emotion recognition, mentalizing, and a total social cognition score from a combination of disparate factors, including clinical diagnosis (healthy controls, subjective cognitive complaints, mild cognitive impairment, Alzheimer’s disease, behavioral variant frontotemporal dementia), demographics (sex, age, education, and country income as a proxy of socioeconomic status), cognition (cognitive and executive functions), structural brain reserve, and in-scanner motion artifacts. Cognitive and executive functions and educational level consistently emerged among the top predictors of social cognition across models. Such non-specific factors showed more substantial influence than diagnosis (dementia or cognitive decline) and brain reserve. Notably, age did not make a significant contribution when considering all predictors. While fMRI brain networks did not show predictive value, head movements significantly contributed to emotion recognition. Models explained between 28–44% of the variance in social cognition performance. Results challenge traditional interpretations of age-related decline, patient-control differences, and brain signatures of social cognition, emphasizing the role of heterogeneous factors. Findings advance our understanding of social cognition in brain health and disease, with implications for predictive models, assessments, and interventions.

## Introduction

Social cognition plays a key role in human interaction, encompassing the mental processes involved in perceiving, interpreting, and responding to others’ social cues (1). The core and most studied components are emotion recognition and mentalizing (2). Emotion recognition conveys the ability to identify how others feel (3). Mentalizing is the capacity to infer others’ mental states, such as their intentions, beliefs, and desires (4). Aging may diminish performance in both processes (5) in association with altered brain signatures (6, 7). Social cognition dysfunction in aging can increase social isolation, loneliness, and vulnerability (8), impacting brain health (9) and quality of life (10). This led standardized tasks of social cognition to be increasingly used in research and clinical contexts to assess the performance of patients with age-related conditions, such as subjective cognitive complaints (SCC), mild cognitive impairment (MCI), and dementia, in comparison to that of healthy controls (HCs) (11–13). However, despite its relevance, several gaps persist in our understanding of the factors that influence social cognition in aging.

One critical problem is the considerable variability observed in social cognition performance among individuals otherwise similar (14), especially in the older ones (5) and in global settings (15, 16). This variability may stem from multiple factors, such as demographic characteristics [sex, age, education (15–19), and socioeconomic status (20)], individual differences in other cognitive abilities (19, 21), and brain reserve [i.e., brain volume (22) and networks (23, 24)]. Relatedly, brain-behavior and brain-phenotype associations often fail to accurately classify individuals with non-stereotypical profiles in terms of clinical presentation, demographics, admixtures, cognition, and brain function (25–28). Functional connectivity-based models usually fail to generalize to diverse samples and are influenced by image acquisition artifacts, particularly in-scanner head motion (25, 29, 30). These issues limit our ability to draw generalized conclusions about social cognition in healthy and pathological aging, hampering the development of a more global agenda and tailored interventions.

**Figure 1:**
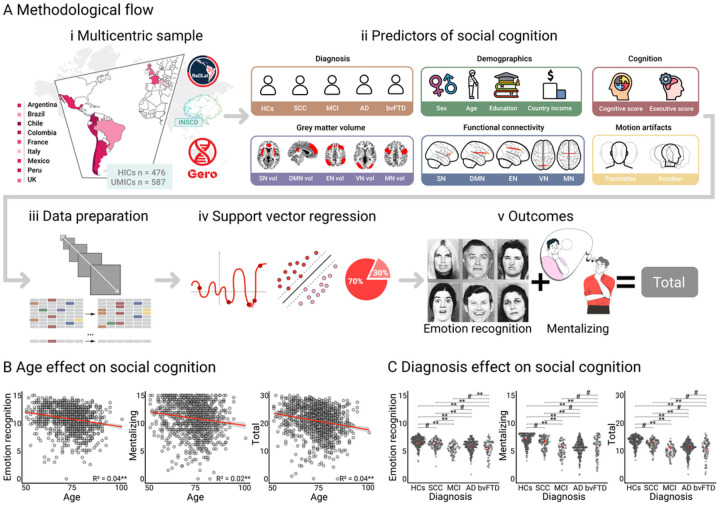
Analysis pipeline and traditional effects of age and diagnosis on social cognition performance. **(A) i**.Participants were recruited from high-income countries (Chile, France, Italy, United Kingdom) and upper-middle-income countries (Argentina, Brazil, Colombia, Peru, Mexico) through the Multi-Partner Consortium to Expand Dementia Research in Latin America (ReDLat), the International Network on Social Condition Disorders (INSCD), and the Geroscience Center for Brain Health and Metabolism (GERO). **ii**.Diagnosis, demographics, cognition, grey matter volume and fMRI resting-state functional connectivity of brain networks, and in-scanner motion artifacts were entered into computational models as predictors of social cognition. **iii**. Data were harmonized across countries (including scale transformation) and missing values were imputed. **iv**. Analysis involved Bayesian optimization with k = 3 cross-validation for tuning the hyperparameters in 70–30 train and test partition and support vector regression models using a bootstrap approach. **v**.Outcome variables were facial emotion recognition, mentalizing, and a social cognition total score from the Mini-Social Cognition and Emotional Assessment (Mini-SEA) battery. **(B)** Age significantly predicted worse performance in emotion recognition, mentalizing, and the total score. The red lines and grey shadings represent the best-fit line for each simple linear regression with 95% confidence bands. **(C)** Participants with MCI, AD, and bvFTD performed significantly worse in social cognition relative to HCs and the SCC group, and participants with bvFTD also performed significantly worse than those with AD in emotion recognition. The red dots and lines display the mean and SD. P values are corrected for multiple comparisons using the Sidak method. AD: Alzheimer’s disease, bvFTD: behavioral variant frontotemporal dementia, DMN: default mode network, EN: executive network,HCs: healthy controls, HICs: high-income countries, MCI: mild cognitive impairment, MN: motor network, SCC: subjective cognitive complaints, SN: salience network, UMICs: upper-middle-income countries, vol: volume, VN: visual network, **P < 0.01, #non-significant.

To address these gaps, we systematically investigated combined predictors of social cognition in older individuals through a multicentric computational approach (see methodological flow in Fig. 1A). We sought to determine whether the typical effects of age (Fig. 1B) and patient-control differences (Fig. 1C) are indeed the primary drivers of performance variability in social cognition tasks. We assembled 1063 participants (> 50 years) from 9 countries to maximize sample diversity. Our outcomes of interest were facial emotion recognition, mentalizing, and a social cognition total score (i.e., the combination of both measures) using a well-characterized assessment (31). The potential predictors comprised the following factors: (a) clinical diagnosis (HCs, SCC, MCI, Alzheimer’s disease-AD, and behavioral variant frontotemporal dementia-bvFTD); (b) demographics [sex (female, male), age (years), education (years), and country income as a proxy of socioeconomic status (high-income countries-HICs, upper-middle-income countries-UMICs) (32)]; (c) cognition [cognitive (33–35) and executive function (36, 37) screening scores]; (d) brain reserve [grey matter volume (38) and functional connectivity strength (39) of the resting-state fMRI networks: salience network-SN (40), default mode network-DMN (41), executive network-EN (42), visual network-VN (43), and motor network-MN (44)]; and (e) in-scanner motion artifacts [average translation and rotation parameters during the resting-state sequence (39)]. The analysis consisted of three distinct model sets. The initial set focused on behavioral data, spanning clinical diagnosis, demographics, and cognition. The second set integrated structural brain reserve factors (grey matter volume) with the previously mentioned behavioral predictors. Lastly, the third set incorporated functional connectivity metrics and motion artifacts, building upon the predictors from both the first and second sets.

We hypothesize that individual differences in social cognition performance will emerge from heterogeneous contributors. More specifically, we anticipate that individuals who are younger in age, healthy, female (15, 16), highly educated (15, 19), from HICs (20), possess strong cognitive and executive abilities (19, 21), and have higher brain reserve (45) will exhibit higher emotion recognition, mentalizing, and total scores. Furthermore, given the emerging evidence on atypical factors influencing cognition (25, 26, 46), we expect that age effects and patient-control differences as traditionally reported in homogeneous, stereotypical samples will not fully account for the observed variability in task performance in a multicentric setting, with other factors increasing the variance explained. Our findings have the potential to advance our understanding of social cognition in aging populations by elucidating the factors that contribute to performance variability in current assessments. This knowledge can inform the development of more robust predictive models and tailored tools to assess and improve social cognition in brain health and age-related diseases.

## Results

### Traditional effects of age and diagnosis on social cognition performance.

Simple linear regression analyses showed that advanced age significantly predicted worse emotion recognition, mentalizing, and the social cognition total score (Fig. 1B and **Table S1**). Linear mixed-effects models (47) controlling for sex, age, education, and country of origin revealed that the diagnosis had a significant effect on emotion recognition (F = 32.88, P < 0.01, η_p_^2^ = 0.12), mentalizing (F = 59.72, P < 0.001, η_p_^2^ = 0.2), and the total score (F = 63.93, P < 0.001, η_p_^2^ = 0.21). Sidak-corrected post-hoc tests showed that HCs and SCC groups outperformed MCI, AD, and bvFTD groups in the three measures, and that individuals with bvFTD performed significantly worse than those with AD in emotion recognition (Fig. 1C). No other significant between-group differences were found.

### Combined predictors of social cognition.

Support vector regression (SVR) models (48) were used to predict social cognition (emotion recognition, mentalizing, and the total score) from the full set of potential predictors. Data were harmonized across countries [through the use of equivalence tables (49, 50), scale transformation, and z-scores estimation], and 170 missing values were imputed using a sklearn iterative imputer with Bayesian ridge regression (51). SVR models were optimized using Bayesian optimization (52) with k = 3 cross-validation for tuning the hyperparameters on training (70%) and testing (30%) folds, with 10 repetitions. Feature selection was performed using backward elimination (53) to identify each model’s top predictors (in order of relevance). To obtain the final models, 1000 optimized SVR regressors were trained and tested for each outcome variable using a bootstrap approach, setting aside median-stratified 30% of the data as test partition. We report the average models’ performance and false discovery rate-corrected P values (statsmodels version 0.13.2). Analyses were performed in the full sample (n = 998, after removing participants with invalid scores) and in subsamples with neuroimaging recordings, including structural MRI (n = 598) and resting-state fMRI (n = 388) sequences.

### Behavioral predictors.

**Figure 2:**
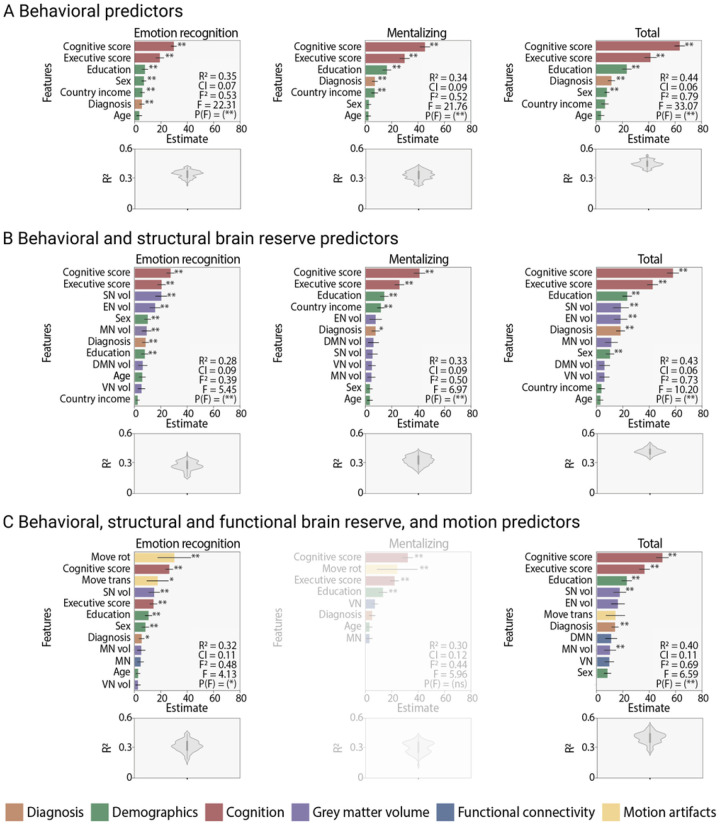
Support vector regression results. **(A)** Models including diagnosis, demographics, and cognition as predictors of social cognition performance. **(B)** Models including one level of brain reserve (grey matter volume) together with behavioral features as predictors of social cognition performance. **C**. Models including resting-state functional connectivity features (brain networks and motion artifacts) as predictors of social cognition performance in addition to behavioral and grey matter volume predictors. Each panel reports the mean and distribution of the coeffi cient of determination R2, 95% CI of R2, Cohen’s *f*^2^, Fisher F test, and largest P value P(F). The translucid panel displays a non-significant model. DMN: default mode network, EN: executive network, MN: motor network, SN: salience network, vol: volume, VN: visual network, *P < 0.05, **P < 0.01.

The first set of models assessed whether behavioral data (clinical diagnosis, demographics, and cognition) were able to predict social cognition (Fig. 2A). The model using emotion recognition as outcome variable was significant [R2 = 0.35, CI (95%) = 0.07, *f*^2^ = 0.53, F = 22.31, P < 0.0001]. The best predictors of emotion recognition were, in order of importance, cognition (β = 29.67, P < 0.0001), executive function (β = 18.98, P < 0.0001), education (β = 7.88, P < 0.0001), sex (β = 7.11, P < 0.0001), country income (β = 5.95, P < 0.0001), and diagnosis (β = 5.51, P < 0.0001). Age was not a significant contributor for emotion recognition (β = 3.53, P > 0.05). Mentalizing was significantly predicted (R2 = 0.34, CI (95%) = 0.09, *f*^2^ = 0.52, F = 21.76, P < 0.0001] by cognition (β = 45.31, P < 0.0001), executive function (β = 29.87, P < 0.0001), education (β = 16.23, P < 0.0001), diagnosis (β = 7.17, P < 0.0001), and country income (β = 7, P < 0.0001). Sex and age were not significant (β = 2.51 and β = 2.34, respectively, both Ps > 0.05). Finally, the social cognition total score was successfully predicted [R2 = 0.44, CI (95%) = 0.06, *f*^2^ = 0.79, F = 33.07, P < 0.0001] by cognition (β = 63.13, P < 0.0001), executive function (β = 41.13, P < 0.0001), education (β = 23.23, P < 0.0001), diagnosis (β = 11.50, P < 0.0001) and sex (β = 8.05, P < 0.0001). Country income and age were not significant (β = 6.55 and β = 3.75, respectively, both Ps > 0.05). Results were similar when assessed without data imputation (**Table S2**), and in the subsamples with structural MRI (**Table S3**) or resting-state fMRI (**Table S4**) data. Taken together, better cognitive and executive functions and higher educational level consistently emerged as the top predictors of social cognition performance, above diagnosis and other demographic characteristics.

### Behavioral and structural brain reserve predictors.

The second set of models included the previous behavioral predictors plus one level of brain reserve (grey matter volume) as predictors of social cognition performance (Fig. 2B). The model predicting emotion recognition was significant [R2 = 0.28, CI (95%) = 0.09, *f*^2^ = 0.39, F = 5.45, P < 0.0001] and included the following features: cognition (β = 27.25, P < 0.0001), executive function (β = 20.37, P < 0.0001), SN volume (β = 20.19, T = 116.11, P < 0.0001), EN volume (β = 15.44, P < 0.0001), sex (β = 9.79, P < 0.0001), MN volume (β = 9.01, P < 0.0001), diagnosis (β = 8.14, P < 0.0001), and education (β = 7.58, P < 0.0001). DMN volume (β = 5.99), age (β = 5.63), VN volume (β = 5.04), and country income (β = 2.12) were not significant (all Ps > 0.05). Mentalizing was also successfully predicted [R2 = 0.33, CI (95%) = 0.09, *f*^2^ = 0.5, F = 6.97, P < 0.0001] by cognition (β = 40.82, P < 0.0001), executive function (β = 25.60, P < 0.0001), education (β = 14.10, P < 0.0001), country income (β = 11.49, P < 0.0001), and diagnosis (β = 7.43, P = 0.03). The EN volume (β = 7.48), the DMN volume (β = 5.87), the SN volume (β = 5.18), the VN volume (β = 4.46), the MN volume (β = 4.31), sex (β = 3.20), and age (β = 3.15, T = 11.07) were not significant (all Ps > 0.05). The social cognition total score was significantly predicted [R2 = 0.43, CI (95%) = 0.06, *f*^2^ = 0.73, F = 10.2, P < 0.0001] by cognition (β = 57.93, P < 0.0001), executive function (β = 42.49, P < 0.0001), education (β = 23.21, P < 0.0001), SN volume (β = 18.38, P < 0.0001), EN volume (β = 18.13, P < 0.0001), diagnosis (β = 18.12, P < 0.0001), and sex (β = 10.13, P < 0.0001). MN volume (β = 11.11), DMN volume (β = 5.95), VN volume (β = 5.88), country income (β = 3.58), and age (β = 2.88) were not significant (all Ps > 0.05). Models including only grey matter predictors were not significant (**Table S5**). Overall, higher cognitive and executive functions and years of education remained among the top predictors of social cognition (together with diagnosis). The higher the grey matter volume of SN, EN, and MN hubs, the larger the contributions to emotion recognition.

### Behavioral, structural and functional reserve, and motion artifacts predictors.

The last set of models included the previous two set of predictors (behavior and grey matter volume) plus functional connectivity and motion artifacts predictors (Fig. 2C). Emotion recognition was significantly [R2 = 0.32, CI (95%) = 0.11, *f*^2^ = 0.48, F = 4.13, P < 0.014] predicted by rotation movements (β = 30.07, P < 0.0001), cognition (β = 26.24, P < 0.0001), translation movements (β = 17.40, P < 0.034), SN volume (β = 14.72, P < 0.034, P < 0.0001), executive function (β = 14.24, P < 0.0001), education (β = 10.49, P < 0.0001), sex (β = 8.10, P < 0.001), and diagnosis (β = 5.22, P < 0.036). MN volume (β = 4.95), MN (β = 4.42), age (β = 2.48), and VN volume (β = 2.41) were not significant (all Ps > 0.05). Mentalizing was not successfully predicted in this model. Finally, the social cognition total score was significantly [R2 = 0.4, CI (95%) = 0.11, *f*^2^ = 0.69, F = 6.59, P < 0.0001] predicted and characterized by cognition (β = 49.85, P < 0.0001), executive function (β = 36.16, P < 0.0001), education (β = 22.67, P < 0.0001), SN volume (β = 17.32, P < 0.0001), diagnosis (β = 13.73, P < 0.0001), and MN volume (β = 9.90, P < 0.0035). EN volume (β = 15.90), translation movements (β = 14.02), DMN (β = 10.76), VN (β = 9.31), and sex (β = 7.95) did not contribute to the model (all Ps > 0.05). Models including functional connectivity and motion features alone (Table S6) and functional connectivity and motion features together with grey matter predictors (i.e., only brain reserve, Table S7) were not significant. Briefly, better cognitive and executive functions, higher education, and more grey matter volume of SN hubs remained among the best predictors of social cognition together with diagnosis. While brain networks did not make significant contributions to the models, higher motion artifacts were associated with emotion recognition.

## Discussion

We investigated the top predictors of social cognition in aging. Two main strengths enabled us to address this issue systematically: (a) the use of a diverse sample of 1063 older individuals from 9 countries, representing a wide range of demographics and socioeconomic contexts, and (b) the development of a multicentric computational approach that thoroughly examined the combined influence of disparate factors. As hypothesized, the combination of heterogeneous factors explained between 28–44% of the variance in emotion recognition and mentalizing tasks, with large effect sizes (*f*^2^ = 39–79). This outcome differs from the comparatively weaker influence of age on social cognition when evaluated independently. Also, while diagnostic differences on social cognition followed the expected pattern, with MCI and dementia groups performing poorer than HCs and SCC groups (11, 54), and bvFTD underperforming AD only in emotion recognition (55), diagnosis was not the primary determinant of performance variability. Specifically, the factors behind higher social cognition across models were higher cognitive and executive functions. Such features had more substantial influence than age (which became a non-significant contributor when assessed in combination), clinical diagnosis, and brain reserve (grey matter volume and functional connectivity). Higher educational level was also among the top predictors in most models. These findings challenge traditional interpretations of age-related decline, patient-control differences, and brain signatures of social cognition, emphasizing the significance of heterogeneous factors. This knowledge has implications for the development of customized predictive models of social cognition in diverse aging populations, allowing more accurate and ethical interpretations. Our results also have implications for the development of tailored social cognition assessment tools and interventions in older adults, ultimately leading to improved brain health and quality of life.

The strong influence of cognitive and executive functions on social cognition performance is consistent with a growing body of evidence suggesting that age-related decline in a wide range of socioemotional paradigms is dependent on task demands (19–21, 56–58). Accurately identifying others’ emotions partially rests on attention allocation, with attentional disturbances influencing misrecognition of emotions and affective symptoms (59). Mentalizing relies on the capacity to inhibit one’s own perspective in favor of adopting that of others, a process that requires executive functions (i.e., working memory and set shifting) (21). Thus, the well-established decrease on these general-purpose abilities in older adults (60) seems to explain social cognition decline. Relatedly, as in previous studies (17, 19), higher education also consistently emerged among the top predictors of social cognition performance. Taken together, cognition and education might represent proxies of the cognitive reserve in aging, namely the ability to cope with brain pathology in order to maintain function (61). While a previous work showed that cognitive reserve was not associated with social cognition in older adults (62), such evidence come from a homogeneous HICs population, potentially failing to capture the diversity of individual differences.

Another non-specific factor that predicted better emotion recognition and mentalizing was country income (HICs). The World Bank country classification (32) represents a national level measure of the socioeconomic background of an individual (i.e., social and monetary wealth or power) (63). Socioeconomic status is known to have robust effects in predicting brain health outcomes in older individuals (64). However, its impact on social cognition and emotional processing has only recently been addressed, pointing to a mediator role of cognitive and executive functions (20). Our results expand this emergent research by revealing a unique contribution of socioeconomic status to social cognition performance. Finally, female sex was associated with improved emotion recognition (but not mentalizing), as previously observed (15, 62). While evidence suggests that women’s advantage in identifying others’ emotions may be a result of gender-role stereotypes (65), more research is needed to determine the underlying mechanisms of such advantage. In summary, our behavioral models suggested that, in addition to cognition and education, socioeconomic status and sex play a significant role in social cognition.

Including brain reserve measures (grey matter volume) in the model architecture did not explain the additional variance in social cognition performance. Moreover, the model that solely utilized grey matter features did not yield predictive value. Cognitive and executive abilities remained the top predictors of emotion recognition, mentalizing, and the total score. Consequently, cognitive reserve may be more relevant than structural brain reserve for social cognition outcomes, potentially reflecting the deployment of active mechanisms (e.g., processing resources or compensation) that facilitate coping with pathology beyond brain size (61). Following cognitive factors, higher grey matter volume of the main hubs of the SN [bilateral insula and anterior cingulate cortex (40)], the EN [bilateral middle frontal and inferior parietal cortex (66)], and the MN [precentral cortex (66)] was associated with better emotion recognition. This finding is consistent with the role of these regions in detecting and attending to salient stimuli (67), as well as in the embodied processing of emotions through mirroring mechanisms (68, 69). Conversely, grey matter volume did not significantly contribute to mentalizing. A possible explanation for this discrepancy could be the higher cognitive demands necessary to mental state inference as opposed to facial emotion recognition, resulting in cognition capturing more variance.

The last set of models showed that fMRI brain network connectivity failed to predict social cognition when combined with behavioral features, brain volume, or when considered independently. Moreover, mentalizing was not significantly predicted in these analyses. In contrast, translation and rotation in-scanner motion artifacts were associated with better emotion recognition (together with cognition, SN volume, education, sex, and diagnosis). Considering the existing evidence on resting-state functional connectivity associations with social cognition [particularly the SN (67) and the DMN (70, 71)], this pattern of results may appear unusual. However, it is becoming increasingly evident that clinical and demographic heterogeneity can hinder the identification of brain-behavior associations (25, 46). Predictive models from homogeneous samples fail to characterize non-stereotypical individuals, particularly from multi-site cohorts (46), with in-scanner motion parameters representing a major source of model failure (25). In brief, brain networks failed to explain social cognition performance, with cognitive and motion features emerging as top predictors in the emotion model, emphasizing the need to consider disparate sources of variability in future studies.

Our work reveals that social cognition components in aging are shaped by heterogeneous factors, adding to recent literature on the sociocultural and demographic determinants of social cognition (14–16). Contrary to mainstream research, our results indicate that age and clinical diagnosis are not the primary drivers of individual differences in social cognition across diverse samples. Although both factors showed the expected effects when assessed independently, such influences attenuate or vanish when heterogeneous factors are considered. Previous works have failed to detect age associations with social cognition after accounting for cognitive (57, 58) and mood [i.e., depression (58)] factors. Older adults might even show improved mentalizing abilities when incorporating education, race, and ethnicity in explanatory models (14). Such evidence raises the question of what current social cognition tasks are actually capturing. It is possible that age-related normal and pathological brain mechanisms become less influential when considered alongside social determinants of brain health and more heterogeneous factors.

These findings have relevant implications for the understanding of social cognition in older adults and the development of customized predictive models. Rather than single constructs, brain-behavior models predict complex profiles (25), and the larger and more heterogeneous the cohorts, the worse they perform (46). Biased models might lead to inaccurate interpretations and decision-making, raising methodological and ethical concerns, with detrimental consequences more likely to affect social disadvantaged populations (72). In addition, our results underscore the need to systematically account for non-specific and contextual influences when assessing social cognition. This is particularly necessary in light of recent recommendations to use standardized social cognition tasks in clinical settings to support patient characterization and differential diagnosis (2, 13, 73). In the same vein, considering the potential of social cognition interventions to improve everyday function (74, 75), accurate predictions turn crucial to advance brain health equity.

Some limitations and additional lines of research must be acknowledged. First, although we used one of the most widely used social cognition assessment (15, 31, 54), it has low ecological validity. Future studies should incorporate more naturalistic stimuli (76). Also, other social cognition components [such as empathy and compassion (77)] should be investigated in older adults. Second, we included only a limited number of countries with unbalanced number of participants, reducing the possibilities for crosscountry interpretations. Global approaches to brain health need larger and more culturally diverse samples. Third, our results should be replicated using other socioeconomic indicators to capture additional inequalities characteristic of regions such as Latin America (e.g., GINI index, human capital index). Relatedly, country-level indexes of socioeconomic status as the one used here should be complemented with measures at the individual or family level (e.g., household income, occupation prestige). Finally, further research should adopt longitudinal designs to understand temporal dynamics in social cognition across brain health and disease and study the causal impact of sociocognitive impairments on real life.

In conclusion, using a multicentric computational approach across three levels of analysis, our findings reveal that social cognition in aging is shaped by a heterogeneous array of cognitive and sociodemographic factors. The most influential predictors were cognitive and executive functions (together with education in most models), which outweighed the impact of age, clinical diagnosis, and brain reserve. The results challenge traditional interpretations of age decline, patient-control differences, and brain signatures of social cognition. We emphasize the need to consider non-specific factors in further studies, with implications for predictive models, assessments, and interventions, aimed at developing more global and inclusive approaches to brain health.

## Materials and Methods

### Participants.

The study comprised 1063 participants between 50 and 98 years (mean age = 71.56, SD age = 8.42, 64.6% women, mean years of education = 12.01, SD years of education = 5). The recruitment was performed across 13 sites in 9 countries, 4 HICs (Chile, France, Italy, United Kingdom, n = 476) and 5 UMICs (Argentina, Brazil, Colombia, Peru, Mexico, n = 587) as classified according to the World Bank (32). The sample included HCs and individuals with different conditions associated with aging (SCC, MCI, AD, and bvFTD, see below). Participants were recruited from different international consortia, including the Multi-Partner Consortium to Expand Dementia Research in Latin America (ReDLat) (78), the International Network on Social Condition Disorders (INSCD) (15), and the Geroscience Center for Brain Health and Metabolism (GERO) (79).

All participants underwent extensive neurological, neuropsychological, and neuropsychiatric examinations comprising semistructured interviews and standardized cognitive assessments. HCs (n = 325) had preserved cognition and no history of neurological or psychiatric conditions. Participants with SCC (n = 145) presented cognitive complaints either self-reported or reported by a knowledgeable informant, scored 0.5 or less on the Clinical Dementia Rating scale (CDR) (80), and had preserved functional abilities (79). The MCI group (n = 96) was composed of participants fulfilling the same criteria as those with SCC but scoring < 22 in the Montreal Cognitive Assessment (MoCA) (34), the most frequently used cut-off to detect MCI (81). Individuals with AD (n = 389) fulfilled the National Institute of Neurological and Communicative Disorders and Stroke-Alzheimer’s Disease and Related Disorders Association (NINCDS-ARDA) criteria (82), were in early and middle stages of the disease, presented memory deficits, and were functionally impaired. Individuals with bvFTD (n = 114) fulfilled the revised Rascovsky criteria (83), were in the early and middle stages of the disease, exhibited prominent behavioral changes, lacked primary language deficits, and had functional impairment. Supporting the clinical diagnosis of neurodegenerative conditions, an analysis of a subsample of participants with available structural MRI data revealed temporal and frontoparietal atrophy in the AD group (46), and fronto-temporo-insular atrophy in the bvFTD group (84) (**Fig. S1** and **Table S8**). Demographic and cognitive information of each participant group is provided in **Table S9**. The IRB of each recruitment site and the Executive Committee of the ReDLat consortium approved this study. All participants signed informed consent in accordance with the Declaration of Helsinki.

### Social cognition assessment.

Participants completed the Mini-Social Cognition and Emotional Assessment (Mini-SEA), a short battery designed to assess two social cognition domains: facial emotion recognition and mentalizing (31). In the facial emotion recognition subtest, participants are asked to identify the emotion being depicted by an individual in 35 different photos from the Ekman series. The following options are provided: fear, sadness, disgust, anger, happiness, surprise, and neutral. Each correct item is given 1 point. The mentalizing subtest consists of an adaptation of the Faux Pas test. Participants are presented with 10 short stories and asked to identify if the protagonist committed an unintended transgression of a social rule (i.e., a faux pas). Each story also includes two control questions to assess general understanding. The maximum score for this subtest is 40 points. The scores of emotion recognition and mentalizing subtests are converted to a score of 15 each and then summed, resulting in a total score of 30, with higher scores representing better performance. From the full sample, 6.11% of participants (n = 65) were removed for lacking a valid score either in the emotion recognition or the mentalizing subtest, resulting in a final sample of 998 individuals.

### Predictors of social cognition.

The set of potential predictors of social cognition included:

**(a) Diagnosis**, HCs, SCC, MCI, AD, and bvFTD.

**(b) Demographics**, sex (female, male), age (years), education (years), and country income (HICs, UMICs) following the World Bank classification (32).

(c)Cognition

**(c.1) Cognitive score**, derived from harmonized scores in the Addenbrooke’s Cognitive Examination III (ACE-III) (35), the Mini-Mental State Examination (MMSE) (33), and the MoCA (34) –see **SI Extended Methods** for details about these tools, **Table S9** for the number of participants assessed with each tool in each group, and Data harmonization section.

**(c.2) Executive score**, derived from harmonized scores in the INECO Frontal Screening (IFS) (37) and the Frontal Assessment Battery (FAB) (36) –see **SI Extended Methods, Table S9**, and Data harmonization section.

(d) Brain reserve

**(d.1) Grey matter volume**, average volume of key hubs of the SN, the DMN, the EN, the MN, and the VN from the Automated Anatomical Labeling (AAL) atlas (85) calculated using voxel-based morphometry (VBM) analysis (see below).

**(d.2) Functional connectivity**, average connectivity strength of the SN, the DMN, the EN, the VN, and the MN calculated via seed analysis of the fMRI resting-state series (see below).

**(e) Motion artifacts**, average translation and rotation movements estimated during the preprocessing of the fMRI sequence.

### Neuroimage acquisition and preprocessing.

This section is reported following recommendations from the Organization for Human Brain Mapping (86). Whole-brain structural 3D T1-weighted and resting-state sequences were obtained for 598 (195 HCs, 91 SCC, 53 MCI, 194 AD, 65 bvFTD) and 388 (125 HCs, 91 SCC, 52 MCI, 82 AD, 38 bvFTD) participants, respectively, across acquisition centers. Demographic and cognitive information of these subsamples are provided in **Table S10** and Table S11. Scanning protocols followed by each center are detailed in **Table S12** and **Table S13**. Structural MRI scans were preprocessed using the DARTEL Toolbox following standard procedures for VBM (38) through the Statistical Parametric Mapping software (SPM12, https://www.fil.ion.ucl.ac.uk/spm/software/spm12/). Functional images were preprocessed using the Data Processing Assistant for Resting-State fMRI toolbox (DPARSF V4.4, http://rfmri.org/DPARSF) following published procedures (39) –see details in **SI Extended Methods**. Six movement parameters (right, forward, up, pitch, roll, yaw) were estimated during realignment to calculate average translation and rotation movements per participant (group statistics are reported in **Table S14**).

### Data harmonization.

To harmonize the available data and increase the number of participants with homogeneous cognitive and executive measures, two procedures were applied. First, cognitive screening measures were harmonized using equivalence tables (49, 50), as previously reported (87). This procedure allows for estimations of MoCA and ACE scores using MMSE scores, and estimations of the MMSE scores using MoCA and ACE scores. As a result, a total of three new converted-harmonized variables were added. Then, the MMSE and MoCA scores were transformed from 0–30 to 0–100 scale and averaged with ACE score to create a single cognitive score per participant (scale 0–100). All participants had a cognitive score. Finally, IFS and FAB scores were also transformed into 0–100 scale and average to obtain a single executive score per participant. In total, 833 participants had an executive score.

Second, we calculated z-scores for demographic (sex, age, education, country income), cognitive (cognitive score, executive score), grey matter, functional connectivity, and motion artifacts variables. For neuroimaging variables, z-scores were estimated using normative data from each fMRI acquisition center according to the following equation:

$$x_{z}=\frac{x-\mu }{s}$$

where:

*x*_*z*_ is the new value,

*x* is the original raw score,

*μ* is the mean score for HCs from the center to which the participant belongs, and

*s* is the standard deviation for HCs from the site or center to which the participant belongs.

### Data imputation.

A sklearn iterative imputer with Bayesian ridge regression (51) was used to impute missing values for age (n = 4), education (n = 2) and executive score (n = 165). This algorithm applies a multivariate imputing strategy modeling a column with missing values as a function of other features and using the estimate for imputation. Each feature is imputed sequentially allowing the usage of prior imputed values on the model that predicts later features. This process is repeated several times, allowing increasingly better estimates of missing values to be calculated as the missing values for each feature are estimated.

### SVR models.

To generate predictions of continuous variables (Mini-SEA emotion recognition, mentalizing, and total scores) from multimodal features (diagnosis, demographics, cognition, brain reserve, motion artifacts), we used SVR models. SVR is a variation of support vector machine which allows linear and non-linear regression. SVR transforms the feature space to establish a hyperplane that best fits the training data, while also minimizing the generalization error on new, unseen data (48). The hyperplane is defined as the set of all points x in the feature space such that:

w⋅x+b=0

where *w* is the weight vector, *b* is the bias term, and · denotes the dot product.

The SVR model seeks to find the weight vector *w* and bias term *b* that satisfy this constraint, while also minimizing the distance between the hyperplane and the training data. The distance is measured using a loss function, typically the ε-insensitive loss:

$$L(y,\hat{y})=max(\left|y-\hat{y}\right|-\epsilon ,0)$$

where is *y* the predicted target value, ŷ and is *ϵ* a small constant that defines the width of the margin around the hyperplane. The loss function penalizes errors that exceed *ϵ*, but ignores errors that fall within *ϵ*.

To find the optimal weight vector *w* and bias term *b*, SVR introduces two slack variables *ξ*_*i*_ and ${\hat{\xi }}_{i}$ for each training example, which allow for violations of the margin and the *ϵ*-insensitive loss, respectively. The optimization problem for SVR is then given by:

Minimize:

$$\frac{1}{2}∥\text{w}{∥}^{2}+\text{C}\left(\sum_{\text{i}=1}^{\text{n}}\left(\xi_{\text{i}}+{\hat{\xi }}_{\text{i}}\right)\right)$$

Subject to:

$${\text{y}}_{\text{i}}-\langle \text{w},\phi \left({\text{x}}_{\text{i}}\right)\rangle \leq \varepsilon +\xi_{\text{i}}\text{ i}=1,\cdots ,\text{n}\;\langle \text{w},\phi \left({\text{x}}_{\text{j}}\right)\rangle -{\text{y}}_{\text{i}}\leq \varepsilon +{\hat{\xi }}_{1}\text{ i}=1,\cdots ,\text{n}\;\xi_{\text{i}}\geq 0,\;{\tilde{\xi }}_{1}\geq 0\text{ i}=1,\cdots ,\text{n}$$

where *C* is a hyperparameter that controls the trade-off between the margin width and the number of violations allowed, and *n* is the number of training examples. The first term in the objective function encourages a wide margin, while the second term penalizes violations of the margin and the *ϵ*-insensitive loss.

SVR can be extended to handle non-linear regression tasks by using a kernel function to map the input data to a higher-dimensional feature space, where the problem may become linearly separable. The optimization problem then becomes:

Minimize:

$$-\frac{1}{2}\sum_{\text{i},\text{j}=1}^{\text{n}}\left(\alpha_{\text{i}}-\hat{\alpha_{\text{i}}}\right)\left(\alpha_{\text{i}}-\hat{\alpha_{\text{i}}}\right)\text{K}\left({\text{x}}_{\text{i}},{\text{x}}_{\text{j}}\right)-\epsilon$$

Subject to:

$$\sum_{\text{i}=1}^{\text{n}}\left(\alpha_{\text{i}}-\hat{\alpha_{\text{i}}}\right)=0\;0\leq \alpha_{\text{i}},\hat{\alpha_{i}}\leq \text{C}$$

where *K* (*x*_*i*_, *x*_*j*_) is the kernel function that computes the inner product between the mapped feature vectors, and *α*_*i*_ and are Lagrange multipliers that determine the importance of each training example in defining the hyperplane. The kernel function allows SVR to learn complex, non-linear relationships between the input features and the target variable.

### Hyperparameter tuning.

A Bayesian optimization (52) with k = 3 cross-validation was applied for tuning the hyperparameters. A Radial Basis Function kernel was used with optimized gamma value. Models with the best hyperparameters were trained on a training sample (70%) and tested in a testing set (30%), with 10 repetitions –**SI Extended Methods**.

### Feature selection.

We used a backward elimination approach (53) to select the most significant predictors for each model. For each iteration, we dropped the predictor with the largest P value until we reached a statistically significant model, a predictor with a P value that became statistically significant, or a model with two predictors.

## Statistical analyses

### VBM analysis.

Using VBM preprocessed structural images, we calculated the average grey matter volume (mL, corrected by total intracranial volume) of 116 regions of the AAL atlas (85) to create grey matter volume indexes of the main hubs of the SN [average of the bilateral anterior cingulum and insula volume (40)], the DMN [average of the bilateral medial frontal and posterior cingulate volume (41)], the EN [average of the bilateral middle frontal and inferior parietal volume (66)], the VN [average of the bilateral occipital volume (66)], and the MN [average of the bilateral precentral volume (66)].

### Functional connectivity analysis.

The functional connectivity strength of the SN, the DMN, the EN, the VN, and the MN was calculated using seed analysis. Two bilateral seeds were placed on cubic regions of interest (voxel size = 7×7×7) for each network: the dorsal anterior cingulate cortex for the SN (40), MNI coordinates 10, 34, 24 and − 10, 34, 24; the posterior cingulate cortex for the DMN (41), MNI coordinates 3,−54, 27 and − 3,−54, 27; the middle frontal gyri for the EN (42), MNI coordinates 30, −2, 62 and − 30, −2, 62; the primary visual cortex for the VN (43), MNI coordinates 8, −92, 8 and − 8, −92, 8; and the primary motor cortex for the MN (44), MNI coordinates 32, − 30, 68 and − 32, −30, 68. The Pearson correlation coeffi cient between the averaged BOLD signal of each pair of seeds and voxels comprised in standard masks (88) typically involved in each resting-state network was used to extract one feature per network for each participant. The statistical significance of the resting-state networks was tested by comparing them with null surrogate models. This approach enables robust statistical evaluations to ensure that the results observed are not obtained by chance but represent a true characteristic of the underlying system (89). The surrogate data technique is based on comparing a particular property of the data (a discriminating statistic) with the distribution of the same property calculated in a set of constructed signals (surrogates) that match the original data set but do not possess the property that is being tested. To this end, we used Fourier transform-based surrogates to recreate the brain’s complex-system dynamics, including uncorrelated and correlated noise, coupling between different brain areas, and synchronization. We found that all the computed resting-state networks were statistically significant against null connectivity (SN: P = 0.02, DMN: P = 0.02, EN: P = 0.03, VN: P = 0.02, MN: P = 0.03), further corroborating our connectivity methods.

### Age effects on social cognition.

Simple linear regression analyses were used to evaluate the predictive value of age on emotion recognition, mentalizing, and the social cognition total score. Analyses were performed in R software. The alpha threshold was set at P < 0.05. Effect size was evaluated with *f*^2^, following Cohen’s criteria (90): stating that 0.02 indicates a small effect, 0.15 indicates a medium effect, and 0.35 indicates a large effect.

### Social cognition performance across diagnostic groups.

Linear mixed-effects models (47) were performed in R to examine between-group differences in emotion recognition, mentalizing, and the total score. Sex, age, and education were entered in the model as covariates of no-interest and participant’s country of origin was entered as a random effect. Post-hoc tests were corrected using the Sidak method. The alpha threshold was set at P < 0.05. Effect size was evaluated with η_p_^2^ (91) where 0.01 indicates a small effect, 0.06 indicates a medium effect, and 0.14 indicates a large effect.

### SVR model estimation And performance assessment.

We trained and tested 1000 optimized SVR regressors for each outcome variable to obtain the final models using a bootstrap approach. We applied P value correction for false discovery rate using statsmodels (version 0.13.2) and set aside median-stratified 30% of the data as test set. To evaluate models’ performance, we used four statistics: the coefficient of determination R2, 95% CI, Cohen’s *f*^2^ (90), Fisher F test, and the largest corrected P values. Outlier results (R2 < IQR − 1.5 * SD and R2 > IQR + 1.5 * SD) were discarded to improve average estimates.

## Data Availability

Anonymized data and code used for study are available on GitHub: https://github.com/AI-BrainLat-team/Global-Mini-SEA. Preprocessed MRI/fMRI data are available on the Open Science Framework: https://osf.io/s754k/?view_only=963c45a837744ff394e993fb320b99ea.
